# MELAS Syndrome with Cardiac Involvement: A Multimodality Imaging Approach

**DOI:** 10.1155/2016/1490181

**Published:** 2016-11-07

**Authors:** Sara Seitun, Laura Massobrio, Anna Rubegni, Claudia Nesti, Margherita Castiglione Morelli, Sara Boccalini, Athena Galletto Pregliasco, Irilda Budaj, Luca Deferrari, Gian Marco Rosa, Fabrizio Montecucco, Alberto Valbusa

**Affiliations:** ^1^Department of Radiology and Interventional Radiology, IRCCS AOU San Martino, IST Genova, Largo Benzi 10, 16100 Genova, Italy; ^2^Clinic of Cardiovascular Disease, University of Genoa, Viale Benedetto XV n6, 16143 Genova, Italy; ^3^Neuromuscular and Molecular Medicine Unit, IRCCS Stella Maris Foundation, Pisa, Italy; ^4^Division of Cardiology, IRCCS AOU San Martino, IST Genova, Largo Benzi 10, 16100 Genova, Italy; ^5^IRCCS AOU San Martino, IST Genova, Largo Benzi 10, 16100 Genova, Italy; ^6^First Clinic of Internal Medicine, Department of Internal Medicine, University of Genoa, Viale Benedetto XV n6, 16143 Genova, Italy

## Abstract

A 49-year-old man presented with chest pain, dyspnea, and lactic acidosis. Left ventricular hypertrophy and myocardial fibrosis were detected. The sequencing of mitochondrial genome (mtDNA) revealed the presence of A to G mtDNA point mutation at position 3243 (m.3243A>G) in *tRNA* ^Leu(UUR)^ gene. Diagnosis of cardiac involvement in a patient with Mitochondrial Encephalomyopathy, Lactic Acidosis, and Stroke-like episodes syndrome (MELAS) was made. Due to increased risk of sudden cardiac death, cardioverter defibrillator was implanted.

## 1. Introduction

Mitochondrial disease is a multisystem disorder with highly heterogeneous clinical pictures and can present at any age [1]. The disorder may affect virtually any organ and cause significant morbidity. The prevalence of mtDNA disease is estimated at 1 : 5000 individuals in Western populations, and 1 : 200 of healthy newborns harbour a potentially pathogenic mtDNA mutation [1]. The m.3243A>G mutation in the MTTL1 gene (*tRNA* ^Leu(UUR)^) is one of the commonest mtDNA mutations and can cause several clinical phenotypes including, such as in the presented case, Mitochondrial Encephalomyopathy, Lactic Acidosis, and Stroke-like episodes syndrome (MELAS) [2–4]. Other common symptoms include seizures, cognitive impairment, muscle weakness and exercise intolerance, sensorineural hearing loss, cardiomyopathy, migraine, bowel dysmotility, and short stature [4]. According to a recent retrospective study, hearing loss and diabetes were the most frequent clinical features, followed by stroke-like episodes [4]. In this context, genetic counseling is an important component of patient diagnosis [4].

## 2. Case Report

**Figure 1:**
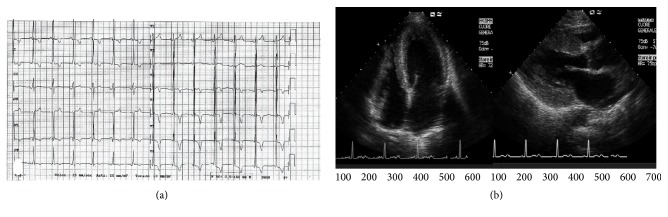
(a) Electrocardiogram showing short PR interval and left ventricular (LV) hypertrophy. (b) Echocardiogram showing concentric, nonobstructive LV hypertrophy.

**Figure 2:**
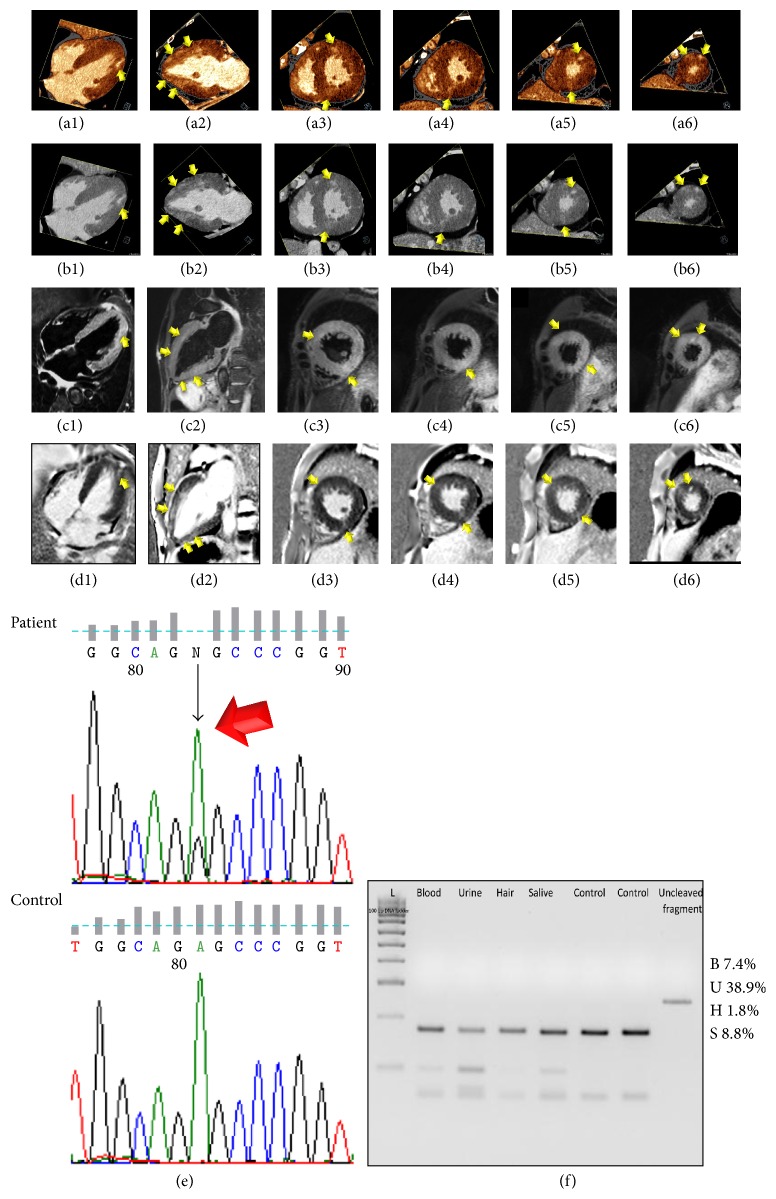
Color-coded (a1–a6) and merged gray-scale (b1–b6) late-enhancement Dual-Energy CT perfusion maps in four-chamber (a1, b1), two-chamber (a2, b2), and short-axis views from base to apex (a3–a6 and b3–b6, resp.) showing the left ventricular (LV) hypertrophy and diffuse, patchy, nonischemic (predominantly intramural), late-enhancement (arrows). T2-STIR MRI imaging (c1–c6) and phase sensitive T1-weighted inversion recovery late-enhancement MRI images (d1–d6) in four-chamber (c1, d1), two-chamber (c2, d2), and short-axis views from base to apex (c3–c6 and d3–d6, resp.) demonstrated diffuse, patchy, nonischemic (predominantly intramural) myocardial edema and late-enhancement consistent with necrosis/fibrosis with a high level of concordance with Dual-Energy CT. (e) Sequence chromatograph of the *tRNA* ^Leu(UUR)^ region flanking the m.3243A>G mutation (arrow) in blood sample from the proband and in a wild type sample (Ctr). (f) PCR-Restriction Fragment Length Polymorphism analysis showed the variable mutant load in patient's peripheral tissues. B: blood; U: urine; H: hair; S: saliva.

A 49-year-old man presented at emergency room with severe chest pain, dyspnea, and metabolic decompensation with lactic acidosis. Personal medical history was also characterized by mild developmental delay, short stature, hearing loss, renal and glycometabolic failure, lactic acidosis, and a family history of sudden death in two maternal relatives. Serial Troponin I was elevated and reached a peak value of 6.8 ucg/L. Laboratory examinations revealed also a significantly increased BNP value (808 ng/L, normal value < 110 ng/L) and glucose level (852 mg/dL, normal value < 110 mg/dL) and a moderate increase in creatinine level (2.3 mg/dL, normal value < 1.3 mg/dL) and lactate value (2.2 mmol/L, normal value < 1.6 mmol/L). Electrocardiogram (ECG) revealed short PR interval and left ventricular (LV) hypertrophy (Figure 1(a)). Echocardiogram showed concentric, nonobstructive LV hypertrophy (Figure 1(b)) with ejection fraction (EF) of 55%, mild aortic and mitral regurgitation, interatrial defect with left to right shunt, and restrictive filling pattern. Urgent cardiac-CT using a second-generation dual-source 128 slices CT scanner (Somatom Definition Flash, Siemens, Erlangen, Germany) revealed the presence of predominantly noncalcified atherosclerotic plaques without significant obstructive coronary stenosis and a severe concentric LV hypertrophy with preserved global systolic function. The global LV myocardial mass was increased to 186 gr/m^2^ (reference range: 70–113 gr/m^2^) with a maximum wall thickness at the anterior basal wall of 20 mm. A small pericardial effusion and a mild hypertrophy of the right ventricle with a maximum wall thickness of 8 mm were demonstrated. After 8 minutes, a late-enhancement acquisition using the dual-energy technique (100/140 kV setting) was performed, showing multifocal areas of late-enhancement (Figures 2(a1)–2(a6) color-coded maps; and Figures 2(b1)–2(b6) merged gray-scale maps, arrows) with a nonischemic pattern (subepicardial and predominantly intramural). Considering the information obtained by clinical history, laboratory testing, and imaging, the patient underwent thorough genetic assessment due to a high index of suspicion for a mitochondrial disorder. A cerebral CT performed as part of the diagnostic work-up, showed atrophy of the cerebellar vermis, basal ganglia calcifications, and focal white matter hypodense lesions consistent with lacunar infarcts. A cardiac Magnetic Resonance Imaging (MRI) study was performed (1.5 T, Aera, Siemens Medical Systems, Erlangen, Germany) after 2-week follow-up of supportive medical therapy showing multifocal areas of myocardial edema on T2-STIR images with a nonischemic distribution (Figures 2(c1)–2(c6), arrows) and a persistent but reduced LV hypertrophy, accounting for a global myocardial mass of 155 gr/m^2^ with a maximum wall thickness at the anterior basal wall of 15.5 mm; the global LV systolic function was still preserved. Multifocal patchy late-enhancement concerning the presence of nonischemic necrosis/fibrosis was demonstrated too (Figures 2(d1)–2(d6), arrows), which correlated with late-enhancement detected by Dual-Energy CT. 48-hour ECG monitoring was performed and neither atrial nor ventricular arrhythmia were detected. However, after considering the increased risk of sudden cardiac death due to the strong family history and the evidence of myocardial fibrosis, primary prophylaxis with implantable cardioverter defibrillator (ICD) was performed. After a lengthy hospital stay with supportive care, the patient was later discharged from the hospital to home asymptomatic and without further complications. During hospitalization and at discharge, the patient did not present with any neurological disturbances requiring antiepileptic medications, antioxidants, and other cofactors [5]. The sequencing of the whole mitochondrial genome (mtDNA) revealed the presence of the A to G mitochondrial DNA (mtDNA) point mutation at position 3243 (m.3243A>G) in *tRNA* ^Leu(UUR)^ gene (Figure 2(e), arrow), with variable mutant load in all the analyzed tissues (Figure 2(f)).

## 3. Discussion

Since muscle contraction and relaxation are active processes which involve energy metabolism and mitochondrial function, an impaired oxidative phosphorylation may in result in cardiac dysfunction. Although the evidence from literature is limited, a recent prospective study demonstrated the presence of LV hypertrophy assessed by echocardiography in 2 of 8 adult patients with MELAS syndrome [6]. Nevertheless, cardiac manifestation (arrhythmias, dilated or hypertrophic cardiomyopathy) was reported to occur in about 38% of patients with MELAS syndrome [7]. To the best of our knowledge, this is the first case of a comprehensive integrated imaging with both CT and MRI in a MELAS syndrome patient with hypertrophic cardiomyopathy. Importantly, cardiac-CT can be useful to exclude obstructive coronary artery disease in the acute evaluation of chest pain patients presenting with normal or low-positive troponin-elevation [8]. According to a recent study [9], the presence of a concentric hypertrophic remodelling pattern is frequently found in MELAS-like patients. Furthermore, late gadolinium enhancement (LGE) at MRI, which reflects expansion of the myocardial interstitium caused by disperse interstitial fibrosis, partial myocardial disarray, and fibrous replacement of irreversibly injured myocytes, was present in 73% of patients (*n* = 8/11) with a diffuse, nonischemic (predominantly intramural), patchy pattern [9], such as in the presented case. Another interesting mechanism involved in the development of the LGE pattern observed in MELAS patients might be vessels leakage due to mitochondrial angiopathy, such as for stroke-like lesions [9]. The vasogenic effect translates into changes in microvascular permeability and extracellular edema formation that may in part contribute to the myocardial LGE [9]. That is in accordance with the presence of multifocal edema at the T2-STIR sequences [9]. In the current case, the reduction of myocardial mass of approximately 17% observed between cardiac-CT and cardiac-MRI was supposed to be related primarily to myocardial edema regression after supportive medical therapy, although different techniques and software applications between the two methods may in part explain that discrepancy.

Myocardial viability imaging by delayed-enhancement Dual-Energy CT is a recently introduced and promising technique that takes advantage of the fact that iodine, the typically used contrast material in CT, shows similar washout kinetics of gadolinium and has a very unique dual-energy absorption characteristic at different X-ray spectra. In this way, by generating color-coded perfusion map of iodine distribution, it is possible to enhance contrast resolution of CT imaging to achieve a better detection of iodine concentration within myocardial fibrotic scar [10]. This has allowed imaging of infarcted myocardium with a high level of concordance between CT and MRI. Similarly to other genetic cardiomyopathies (including hypertrophic cardiomiopathy), LGE detection may play an additional prognostic role in the risk stratification of MELAS patients since LGE has been related to worsening of LV function and adverse outcome [9, 11].

The current study highlights the importance of multimodality imaging with echocardiography, cardiac-CT, and cardiac-MRI if there is any suspicion of cardiac involvement associated with a rare neurometabolic disorder. In such cases, integrated information derived by CT and MRI regarding the presence and distribution of coronary atherosclerosis, myocardial edema, and late-enhancement may have important therapeutic and prognostic consequences.
